# Genome assembly of *Erythrophleum Fordii*, a special “ironwood” tree in China

**DOI:** 10.1186/s12863-023-01176-9

**Published:** 2023-11-28

**Authors:** Chang-Yu Wen, Ju-Yu Lian, Wei-Xiong Peng, Zheng-Feng Wang, Zhi-Gang Yang, Hong-Lin Cao

**Affiliations:** 1 Guangdong Forestry Survey and Planning Institute, Guangzhou, 510520 China; 2 Guangdong Provincial Key Laboratory of Applied Botany, South China Botanical Garden, Chinese Academy of Sciences, Guangzhou, 510650 China; 3 Key Laboratory of Vegetation Restoration and Management of Degraded Ecosystems, South China Botanical Garden, Chinese Academy of Sciences, Guangzhou, 510650 China; 4 South China National Botanical Garden, Guangzhou, 510650 China

**Keywords:** De novo assembly, Genome feature, Genome survey, Gene annotation, RNA-seq

## Abstract

**Objectives:**

*Erythrophleum* is a genus in the Fabaceae family. The genus contains only about 10 species, and it is best known for its hardwood and medical properties worldwide. *Erythrophleum fordii* Oliv. is the only species of this genus distributed in China. It has superior wood and can be used in folk medicine, which leads to its overexploitation in the wild. For its effective conservation and elucidation of the distinctive genetic traits of wood formation and medical components, we present its first genome assembly.

**Data description:**

This work generated ~ 160.8 Gb raw Nanopore whole genome sequencing (WGS) long reads, ~ 126.0 Gb raw MGI WGS short reads and ~ 29.0 Gb raw RNA-seq reads using *E. fordii* leaf tissues. The *de novo* assembly contained 864,825,911 bp in the *E. fordii* genome, with 59 contigs and a contig N50 of 30,830,834 bp. Benchmarking Universal Single-Copy Orthologs (BUSCO) revealed 98.7% completeness of the assembly. The assembly contained 471,006,885 bp (54.4%) repetitive sequences and 28,761 genes that coded for 33,803 proteins. The protein sequences were functionally annotated against multiple databases, facilitating comparative genomic analysis.

## Objective

*Erythrophleum* is a genus in the Fabaceae family and contains only about 10 acceptable species in total [1]. However, these species are widely distributed throughout the world, with six found in Africa, three in Asia and one in Australia, displaying a clearly disjunct distribution pattern. All *Erythrophleum* species grow as medium-sized or large trees, up to tens of metres [2–4]. *Erythrophleum* species have high-quality wood that is hard, dense, heavy and tough and contains a variety of secondary metabolites (e.g. alkaloids, terpenoids and flavonoids) in different parts (leaf, bark, stem or seed), which are valuable for the treatment of many illnesses [1, 4–7]. Therefore, *Erythrophleum* species are threatened due to their hardwood and/or biomedical properties in different distribution areas [2–4, 6, 7]. In addition to timber and medicinal uses, *Erythrophleum* species can be used as ornamental and agroforestry trees [8, 9].

*Erythrophleum fordii* Oliv. is the only species of this genus distributed in China [10]. Except for China, *E. fordii* is also found in Vietnam. In both countries, it is best known for its superior wood, which has a highly condensed lignin structure, leading to its hardness, heaviness and durableness [11]. *Erythrophleum fordii* is also a medicinal plant containing various bioactive components [1, 12–14] and a high alkaloid content [1]. Some triterpenoids in *E. fordii* are species specific [1]. Due to its high economic value, it has been overexploited in history in both China and Vietnam, making it endangered in the wild [3, 10, 11]. For endangered species, contiguous, accurate and annotated genome assemblies greatly enhance their conservation [15]. Therefore, we present here the first fully annotated *E. fordii* genome for its effective conservation in the future. The genome will also help elucidate distinctive genetic traits related to wood formation and secondary metabolites in *E. fordii*, aiding in the molecular breeding of trees.

## Data description

Leaf samples from one *E. fordii* individual planted in the South China Botanical Garden were collected. After total RNA and genomic DNA were extracted from the samples, three sequencing libraries were conducted for the whole genomic and transcriptomic sequencing. The Nanopore PromethION sequencer was used for long-read whole genomic sequencing (WGS), and the MGI DNBSEQ-T7 sequencer for short-read WGS and RNA-seq under 150 bp paired-end mode. After sequencing, different programmes were performed for analysis and default parameters were used unless otherwise mentioned.

Sickle v1.33 [16] was used to trim the WGS short reads with the parameter “-q 30 -l 80”. The trimmed reads were used to estimate the *E. fordii* genome size with KmerGenie v1.7044 [17] using the parameter of “-k 141 --diploid”. Porchop v0.2.4 [18] was used to trim the adapters for WGS long reads with the parameter “--check_reads 500000”. The reads were then filtered by ontbc v1.1 [19] with the parameters of “-min_score 7 -min_length 10000.” The filtered long reads were used to assemble the assembly using NextDenovo v2.3.1 [20]. Pseudohaploid [21] and Purge_Dups v1.2.6 [22] were used to remove duplicated sequences in the assembly. The assembly was further polished by racon v1.5.0 [23], hapo-G v1.3.2 [24] and polypolish v0.5.0 [25]. In the steps using racon and hapo-G, they were each run for two rounds. The completeness of the assembly was assessed by BUSCO v5.4.6 [26] using the Eudicots odb10-2020-09-10 database.

The assembly was parsed through RED v2.0 [27] and EDTA v2.1.0 [28] to identify repeat sequences, and the repeat regions were subsequently soft-masked. The genes were first predicted by braker v.2.0 [29] using both transcriptome data and reference protein sequences (Data file 1) [30]. The braker results were then integrated into Funannotate pipeline v1.8.16 [31] to obtain the non-redundant gene set. The performance of Funannotate gene prediction included three steps: “train”, “predict” and “update”. In each step, the parameter of “--max_intronlen 1000000” was used. In the “predict” step, additional parameters of “--busco_seed_species arabidopsis --organism other --busco_db embryophyta” were used. The predicted genes were functionally annotated against multiple databases using the “funannotate annotate” command in the Funannotate pipeline.

Three sequencing libraries produced ~ 126.0 Gb raw data for WGS short read sequencing (Data file 2) [32], ~ 160.8 Gb for WGS long read sequencing (Data file 3–7) [33–37] and ~ 29.0 Gb for RNA-seq (Data file 8) [38]. The estimated genome size by KmerGenie was 853,550,132 bp. The genome assembly measured 864,825,911 bp with 59 contigs (N50 = 30,830,834 bp) (Data file 9) [39] and a BUSCO completeness of 98.7% (Data file 10) [40]. Repeat prediction by RED and EDTA identified 376,075,788 bp (43.5%) (Data file 11) [41] and 417,133,422 repetitive sequences (48.2%) (Data file 12) [42], respectively. Their combination was 471,006,885 bp, accounting for 54.4% of the genome (data file 13) [43]. A total of 28,761 genes that coded for 33,803 proteins were predicted (Data files 14–16) [44–46] and their annotation was shown in Data files 17 and 18 [47, 48].

## Limitations

The continuousness of the assembled genome could be further improved using ultra-long Nanopore sequencing and Hi-C data.

**Table 1:** Overview of all data files/data sets

Label	Name of data file/data set	File types (file extension)	Data repository and identifier (DOI or accession number)
Data file 1	Table 1 Species with their protein sequences used for gene prediction	Table (.xlsx)	Figshare, 10.6084/m9.figshare.24303265.v1 [30]
Data file 2	Raw WGS short reads	Fastq file (.fastq)	NCBI Sequence Read Archive, https://identifiers.org/ncbi/insdc.sra:SRR26105794 [32]
Data file 3	Raw WGS long reads	Fastq file (.fastq)	NCBI Sequence Read Archive, https://identifiers.org/ncbi/insdc.sra:SRR26143820 [33]
Data file 4	Raw WGS long reads	Fastq file (.fastq)	NCBI Sequence Read Archive, https://identifiers.org/ncbi/insdc.sra:SRR26143821 [34]
Data file 5	Raw WGS long reads	Fastq file (.fastq)	NCBI Sequence Read Archive, https://identifiers.org/ncbi/insdc.sra:SRR26143822 [35]
Data file 6	Raw WGS long reads	Fastq file (.fastq)	NCBI Sequence Read Archive, https://identifiers.org/ncbi/insdc.sra:SRR26152992 [36]
Data file 7	Raw WGS long reads	Fastq file (.fastq)	NCBI Sequence Read Archive, https://identifiers.org/ncbi/ insdc.sra:SRR26152993 [37]
Data file 8	Raw RNA reads of leaf tissues	Fastq file (.fastq)	NCBI Sequence Read Archive, https://identifiers.org/ncbi/insdc.sra:SRR26075053 [38]
Data file 9	Assembled genome	Fasta file (.fasta)	NCBI Nucleotide, https://identifiers.org/nucleotide:JAVQMF000000000.1 [39]
Data file 10	BUSCO assessment of the assembly	Text (.txt)	Figshare, 10.6084/m9.figshare.24303397.v1 [40]
Data file 11	Repetitive sequences predicted by RED	Text file (.bed)	Figshare, 10.6084/m9.figshare.24304657.v1 [41]
Data file 12	Repetitive sequences predicted by EDTA	Gff3 file (.gff3)	Figshare, 10.6084/m9.figshare.24303487.v1 [42]
Data file 13	Repetitive sequences combined by RED and EDTA	Text file (.bed)	Figshare, 10.6084/m9.figshare.24305008.v1 [43]
Data file 14	Predicted gene	Gff3 file (.gff3)	Figshare, 10.6084/m9.figshare.24305032.v1 [44]
Data file 15	Predicted genes - nucleotide sequences	Fasta file (.fasta)	Figshare, 10.6084/m9.figshare.24305245.v1 [45]
Data file 16	Predicted genes - translated sequences	Fasta file (.fasta)	Figshare, 10.6084/m9.figshare.24305251.v1 [46]
Data file 17	Gene annotation using GO, Pfam, interPro and UniProt, dbCAN, MEROPS and SignalP databases	Text (.txt)	Figshare, 10.6084/m9.figshare.24305284.v1 [47]
Data file 18	Gene annotation from eggNOG-mapper analysis	Text (.txt)	Figshare, 10.6084/m9.figshare.24305290.v1 [48]

## Data Availability

Raw sequenced reads have been uploaded to the NCBI Sequence Read Archive under accession number SRR26105794 for short-WGS sequencing reads [32], SRR26143820, SRR26143821, SRR26143822, SRR26152992 and SRR26152993 for long-WGS reads [33–37], SRR26075053 for RNA-seq reads [38], and JAVQMF000000000 for the assembled genome [39]. Please further see Table 1 for details and references [30, 41–48] of the results of the annotations submitted to figshare.
